# Endoscope-Assisted Versus Conventional Posterior Fossa Decompression with Duraplasty for Chiari I Malformation: A Single-Center Comparative Study

**DOI:** 10.3390/medicina62071285

**Published:** 2026-07-03

**Authors:** Mahmut Çamlar, Umut Tan Sevgi, Mustafa Eren Yüncü, Abdullah Bozoklar, Nevzat Semih Parlak, Çağlar Türk, Meryem Merve Ören Çelik, Ali Karadağ

**Affiliations:** 1 Department of Neurosurgery, University of Health Sciences, İzmir City Hospital, Izmir 35540, Türkiye; umuttan1995@gmail.com (U.T.S.); yuncueren@gmail.com (M.E.Y.); drabdullahbozoklar@gmail.com (A.B.); nevsemih@gmail.com (N.S.P.); caglarturk83@gmail.com (Ç.T.);; 2 Department of Public Health, İstanbul Faculty of Medicine, İstanbul University, Istanbul 34093, Türkiye; meryemerve@gmail.com

**Keywords:** Chiari I malformation, Chicago Chiari Outcome Scale, posterior fossa decompression with duraplasty, endoscopic surgery

## Abstract

*Background and Objectives*: Endoscope-assisted posterior fossa decompression with duraplasty (PFDD) is a minimally invasive alternative treatment for Chiari I malformation; however, its comparative effectiveness remains unclear. Therefore, this study aimed to compare the outcomes of conventional open decompression with those of endoscope-assisted minimally invasive decompression combined with duraplasty to assess the balance between limited surgical exposure and associated technical challenges. *Materials and Methods*: This retrospective single-center study compared 22 patients who underwent endoscope-assisted PFDD with a historical cohort of 16 patients treated with conventional open PFDD. Patients with C1–2 instability, prior craniovertebral surgery, or concomitant pathology requiring an alternative surgical strategy were excluded. The clinical outcomes, radiological findings, surgical variables, and complications were analyzed. *Results*: Clinical improvement, overall recovery, and 3-month Chicago Chiari Outcome Scale (CCOS) scores were comparable between the groups. The endoscopic group had higher CCOS scores at discharge. Syrinx resolution rates were similar, whereas postoperative cisterna magna expansion was more limited in the endoscopic cohort. The endoscopic approach was associated with a significantly shorter incision length and earlier mobilization. The rates of complications, including pseudomeningocele, cerebrospinal fluid fistula, and wound infection, did not differ significantly between the groups. *Conclusions*: Endoscope-assisted PFDD may be a less invasive alternative with comparable short-term clinical and radiological outcomes. Despite the technical challenges related to a limited working corridor, it can be considered a feasible option for selected patients.

## 1. Introduction

Chiari malformation type I (CM-I) is characterized by caudal herniation of the cerebellar tonsils through the foramen magnum, resulting in impaired cerebrospinal fluid (CSF) dynamics at the craniovertebral junction, and is frequently associated with syringomyelia [1]. In symptomatic patients, posterior fossa decompression (PFD) with duraplasty remains the standard surgical treatment for achieving adequate neural decompression and the restoration of CSF flow [2].

Recently, minimally invasive, endoscope-assisted, and full endoscopic techniques have gained increasing interest. These techniques offer smaller incisions, reduced soft tissue disruption, and greater patient acceptance. However, they require working through a limited surgical corridor, which makes duraplasty technically demanding, particularly for achieving a reliable watertight closure [3,4]. Despite growing adoption of these techniques, direct comparisons with conventional open surgery under similar operative conditions remain limited [5]. Therefore, this single-center study compared the outcomes of traditional open decompression and endoscope-assisted minimally invasive decompression with duraplasty to evaluate the tradeoff between reduced surgical exposure and technical constraints.

## 2. Materials and Methods

### 2.1. Study Design

This retrospective single-center observational study evaluated patients with CM-I who underwent endoscope-assisted posterior fossa decompression with duraplasty (PFDD) (with C1 laminectomy). The outcomes of this cohort were compared with those of a previously published institutional series of patients treated with conventional open PFDD [6], which served as a historical control group. Only patients who underwent PFDD (with C1 laminectomy) were included in both cohorts to ensure methodological consistency.

### 2.2. Patient Selection

The patients were identified using an institutional surgical database. All consecutive patients who underwent PFDD for CM-I between 1 December 2023, and 10 January 2025, were included. Inclusion criteria consisted of radiologically confirmed CM-I, defined as cerebellar tonsillar descent of ≥5 mm below the foramen magnum, and the presence of Chiari-related symptoms, with or without syringomyelia. Patients were excluded if they had a history of prior craniovertebral junction surgery, Chiari malformation types other than type I, concomitant pathologies requiring alternative surgical strategies, such as tumors, infections, or craniocervical instability, or incomplete clinical or radiological data. All patients were evaluated and deemed surgical candidates based on consistent institutional criteria.

### 2.3. Preoperative Evaluation

All patients underwent standardized preoperative clinical and radiological evaluations. Magnetic resonance imaging (MRI) of the brain and entire spinal axis was performed in all patients to confirm the diagnosis, assess the degree of tonsillar descent, and evaluate the presence and extent of syringomyelia. Computed tomography was routinely performed to evaluate the osseous anatomy of the craniovertebral junctions. Additionally, flexion–extension lateral radiographs were routinely obtained to exclude significant C1–C2 instability. Additional radiological parameters, including the cisterna magna status and relevant anatomical features, were documented and used for surgical planning. All the procedures were performed electively.

### 2.4. Surgical Technique

All procedures were performed under general anesthesia with endotracheal intubation. The patients were positioned prone with the head in slight flexion to optimize exposure of the craniovertebral junction. The level of the skin incision was adjusted according to the individual anatomy. The occipital angle was considered. In patients with a steeper angle, the incision was placed more caudally, whereas in flatter configurations, it was positioned more cranially. The target level was confirmed intraoperatively by fluoroscopy.

**Figure 1:**
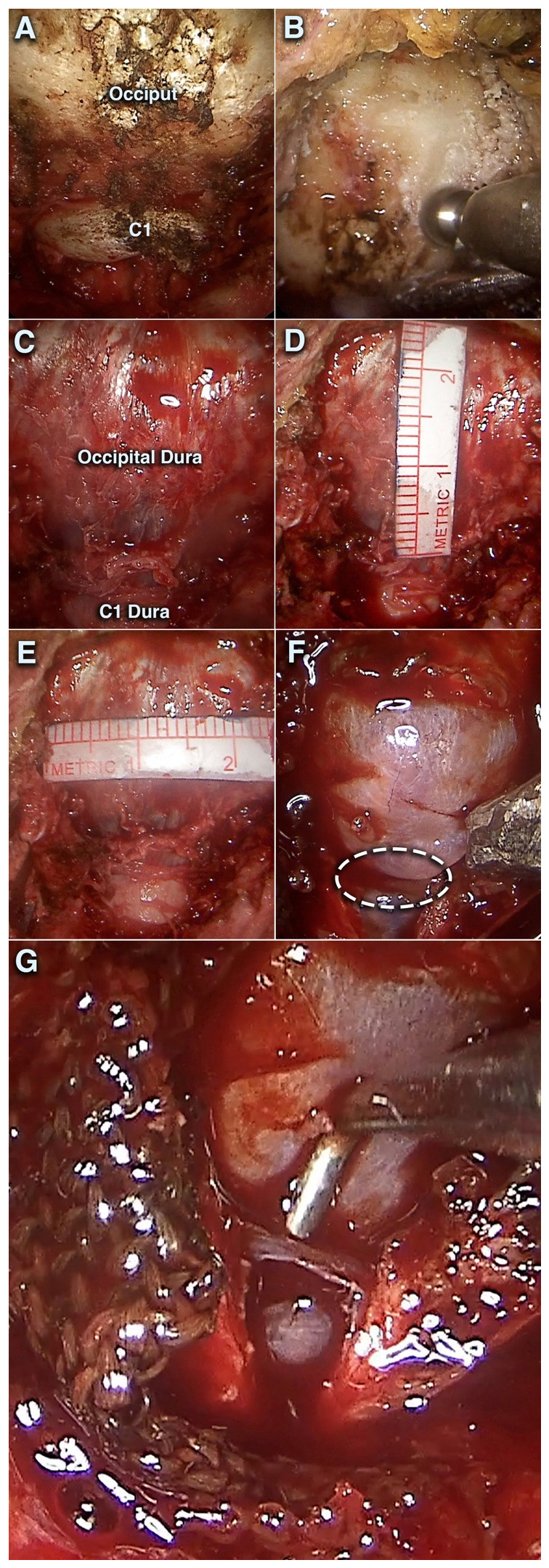
Endoscopic posterior fossa decompression procedure. (**A**) Exposure of the occipital bone and posterior arch of C1 following soft tissue dissection. (**B**) Suboccipital craniectomy performed using a high-speed drill. (**C**) Exposure of the occipital dura and C1 dura after completion of bony decompression. (**D**,**E**) Intraoperative measurements demonstrating the vertical (**D**) and lateral (**E**) dimensions of the craniectomy. (**F**) After dural opening, an intact arachnoid membrane is visualized; a constricting fibrous band limiting arachnoid expansion is highlighted (dashed circle). (**G**) Sharp release of the constricting band with preservation of the arachnoid membrane, allowing restoration of subarachnoid space.

In the endoscope-assisted group, an approximately 2.5 cm midline skin incision was made. Dissection was strictly performed along the midline, following the avascular plane. Subperiosteal exposure was performed until the inferior occipital bone and the posterior arch of C1 were visualized. The exposure was limited superiorly once the inion was identified (Figure 1A).

The procedure was performed using an endoscope-assisted technique. The endoscope was held by the primary surgeon in the non-dominant hand, allowing simultaneous visualization and manipulation within a limited working corridor. In selected cases, angled endoscopes were used to improve visualization, particularly in patients with unfavorable cranial angles (Figure 1B).

Limited suboccipital craniectomy and C1 laminectomy were performed using a high-speed drill and Kerrison rongeur. Bone removal was approximately 2–2.5 cm in diameter and extended laterally along the posterior arch of C1 until the limits of the venous plexus were encountered (Figure 1C–E).

Hemostasis was achieved during the extradural phase using bipolar coagulation and gentle compression, particularly in the areas of venous plexus bleeding before the dural opening.

After bony decompression, the posterior atlanto-occipital membrane and constricting dural band were removed (Figure 1F,G). The dura mater was opened in a Y-shaped fashion. The arachnoid membrane was preserved when possible. Conversion to an open microsurgical approach was regarded as a safety option in cases of uncontrolled bleeding, marked arachnoid disruption, high-flow CSF leakage, or inability to achieve acceptable dural reconstruction through the endoscopic corridor.

Intraoperative Doppler ultrasonography was used to assess the CSF flow dynamics. No additional intradural manipulations were performed when adequate CSF pulsation was observed. In cases in which CSF pulsation remained insufficient, the operative field was inspected for constricting fibrous bands beneath the dura and overlying the cisterna magna arachnoid. When such bands were present, a blunt hook was gently introduced between the band and the arachnoid, and the band was sharply released while preserving the arachnoid membrane. No endoscopic intracisternal maneuvers, tonsillar coagulation, obex exploration, or arachnoid dissection were performed (Video S1).

Duraplasty was performed using an onlay xenograft (DuraGen, Integra LifeSciences, Princeton, NJ, USA). After graft placement, the head position was returned to a neutral alignment, and a fibrin sealant (Tisseel, Takeda Pharmaceutical Company, Lexington, MA, USA) was applied. The wound was closed in the anatomical layers, and a single soft drain was placed.

### 2.5. Outcome Measures

#### 2.5.1. Clinical Outcomes

Clinical outcomes were evaluated based on changes in presenting symptoms and neurological findings at follow-up. Clinical improvement was categorized as no, mild, moderate, or marked improvement according to the extent of symptom resolution. Functional outcome was assessed using the Chicago Chiari Outcome Scale (CCOS) [7] at discharge and at 3 months postoperatively. A CCOS score of 13–16 indicated a favorable outcome. Postoperative complications, including pseudomeningocele, cerebrospinal fluid (CSF) fistula, and wound infection, were recorded.

#### 2.5.2. Radiological Outcomes

Radiological outcomes were evaluated using postoperative MRI. Syringomyelia was classified as disappeared, reduced, unchanged, or newly developed based on a comparison with preoperative imaging. Reduction in the syrinx was defined as a decrease in size on postoperative MRI, whereas complete disappearance was defined as the absence of a visible syrinx cavity. The cisterna magna status was categorized as absent, minimal, or wide [6].

### 2.6. Statistical Analysis

Continuous variables were expressed as the mean ± standard deviation and compared using Welch’s *t*-test. Categorical variables were analyzed using the chi-square or Fisher’s exact test, as appropriate, based on the expected cell counts. Statistical significance was set at *p* < 0.05. Standardized mean differences (SMDs) were calculated to assess baseline balance. Exploratory adjusted sensitivity analyses were also performed for selected outcomes, with surgical approach as the main independent variable.

## 3. Results

### 3.1. Baseline Characteristics

The baseline patient characteristics are summarized in Table 1. The two groups were comparable. However, neck/occipital pain was more frequent in the endoscopic group, while motor deficits were more common in the conventional group. Additionally, preoperative obex visibility was higher in the endoscopic cohort. SMD analysis showed baseline imbalance in sex, headache, neck/occipital pain, motor deficit, and preoperative obex visibility.

### 3.2. Surgical Variables

**Figure 2:**
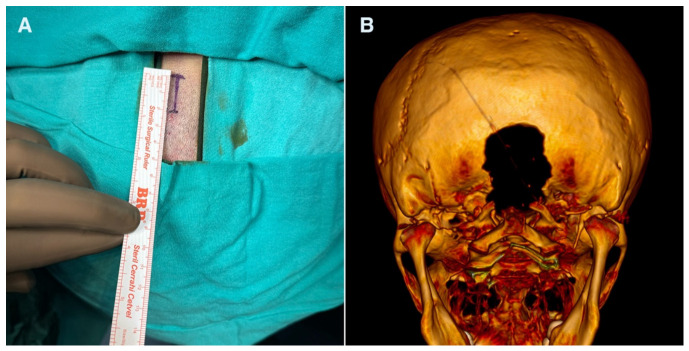
(**A**) Skin incision measuring approximately 2 cm. (**B**) Postoperative three-dimensional CT reconstruction demonstrating the extent of bony decompression.

Surgical variables differed significantly between the two groups. The mean incision length was markedly shorter in the endoscopic group than in the conventional group (2.45 ± 0.50 cm vs. 6.06 ± 0.83 cm, *p* < 0.001). Additionally, patients in the endoscopic cohort achieved earlier postoperative mobilization, with a significantly shorter time to ambulation (0.5 ± 0.5 days vs. 1.5 ± 0.8 days, *p* < 0.001). In addition, the length of hospital stay was significantly shorter in the endoscopic group than in the conventional group (3.0 ± 3.0 days vs. 5.2 ± 2.0 days, *p* = 0.013) (Table 2, Figure 2). No endoscope-assisted procedure required conversion to an open microsurgical approach.

### 3.3. Outcomes

The clinical and radiological outcomes were largely comparable between the two groups. No significant differences were observed in the distribution of clinical improvement, overall recovery, or 3-month CCOS scores. However, the CCOS score at discharge was higher in the endoscopic group.

Radiological outcomes showed no significant differences in postoperative syrinx status. In contrast, postoperative cisterna magna status differed significantly between the groups (Table 2).

Exploratory adjusted sensitivity analyses showed a similar pattern, with early recovery measures remaining favorable in the endoscopic group, whereas 3-month CCOS, overall recovery, complications, and syrinx outcomes remained non-significant; details are provided in Supplementary Tables S1–S3.

### 3.4. Complications

The postoperative complication rates were comparable between the two groups, with no significant differences in the rates of pseudomeningocele, CSF fistula, or wound infection (Table 2). Of the 10 pseudomeningocele cases in the endoscopic group, six were associated with intraoperative arachnoid injury during dural opening, and four of these developed CSF leaks. In the other four cases, pseudomeningocele was observed postoperatively despite the absence of a visible intraoperative CSF leak.

## 4. Discussion

In this study, the endoscopic approach was associated with a less invasive surgical profile, as reflected by a smaller incision size and earlier postoperative mobilization, while providing comparable short-term clinical outcomes, complication rates, and syrinx resolution. Notably, although radiological differences were observed in postoperative cisterna magna expansion, they did not translate into measurable differences in clinical recovery.

Minimally invasive surgery aims to achieve adequate decompression while minimizing surgical morbidity [8,9]. In both conventional and endoscopic approaches, the primary goal is to provide maximal benefit with minimal harm. Endoscopic techniques are associated with less muscle dissection; consistent with the findings of previous studies, the incision length was significantly shorter in the endoscopic group in this study [3,5,8,10]. Shorter skin incisions are associated with less postoperative pain, faster recovery, and improved cosmetic outcomes.

In the present cohort, patients in the endoscopic group demonstrated earlier mobilization, and this difference is likely multifactorial. However, although the endoscopic approach uses a smaller skin incision, the amount of subperiosteal muscle stripping required for bony decompression is largely similar in both techniques. This finding suggests that the shorter time to ambulation may be related less to the extent of the subperiosteal dissection and more to the smaller incision and reduced direct muscle and soft tissue injuries. Therefore, patients in the endoscopic group may have experienced less postoperative pain and lower resistance to mobilization, making earlier ambulation more feasible [11,12]. Reduced pain may also have decreased opioid requirements and their associated side effects, such as nausea, dizziness, and sedation, which may further contribute to delayed ambulation [13].

The primary aim of surgery for CM-I is to relieve neural structure compression at the craniovertebral junction and restore normal CSF dynamics [14]. In this study, duraplasty was performed in both groups, and both approaches achieved this fundamental objective, as the overall clinical recovery rates were similar between the conventional and endoscopic groups. However, when evaluated in terms of recovery kinetics, the endoscopic approach offered an early postoperative advantage.

These results were also reflected in the CCOS findings. CCOS scores at discharge were significantly higher in the endoscopic group than in the conventional group. However, CCOS scores had become more similar between the two groups by the 3-month follow-up. Although CCOS is a provider-based and partly subjective measure with limited sensitivity, it still offers a practical multidimensional framework for outcome assessment. In a comparative study, Gunerhan et al. reported shorter hospital stays in the endoscopic group than in the conventional group [5]. The findings of this study are consistent with this observation, as higher CCOS scores at discharge in the endoscopic group suggest a more favorable early postoperative recovery.

These findings suggest that the minimally invasive corridor provided by the endoscopic technique may facilitate faster early functional recovery, whereas the long-term effects of neural decompression may become comparable over time between the two techniques. However, symptom resolution after Chiari surgery may vary considerably among patients, and these observations should be interpreted cautiously until they are confirmed in larger cohorts [15,16].

Radiologically, a wide postoperative cisterna magna was observed in 75% of patients in the conventional group, whereas this rate was only 27.3% in the endoscopic group. A large proportion of patients in the endoscopic group had only a minimal neocisterna (45.5%) or no visible neocisterna (27.3%). Although postoperative cisterna magna volume may influence clinical improvement, this remains controversial [4,6,17]. For example, despite a significant postoperative increase in CSF cistern volume in a pediatric Chiari I cohort, this volumetric enlargement did not correlate with clinical improvement, as assessed by CCOS [18]. In this series, despite the absence of a wide cisterna magna in many patients in the endoscopic group, the similar rates of syrinx regression between the groups and comparable clinical outcome scores suggest that this radiological difference may not necessarily indicate inadequate decompression. Furthermore, intraoperative Doppler ultrasonography may be helpful in predicting the restoration of adequate CSF flow. Collectively, these findings suggest that rather than creating a large cisterna magna, achieving a cistern sufficient to allow CSF flow may be adequate for both clinical improvement and radiological benefits, including syrinx reduction. However, this interpretation should be confirmed in larger studies.

It should also be noted that the endoscopic approach primarily reduced the skin incision and soft-tissue corridor, it does not necessarily mean insufficient osseous decompression. Since operative corridor is widened in a cone-shaped fashion after the limited skin entry, it still allows adequate suboccipital decompression and C1 laminectomy. Therefore, the smaller postoperative cisterna magna observed in some endoscopic cases may be caused by the geometry of the duraplasty, arachnoid preservation, and limited soft-tissue expansion rather than insufficient osseous decompression alone. Nevertheless, the 3-month follow-up remains too short to exclude delayed clinical failure, recurrent symptoms, persistent CSF-flow obstruction, or late syrinx progression. Thus, the present findings should be interpreted as short-term feasibility data, and longer follow-up is required to determine whether limited cisterna magna expansion has any medium- or long-term clinical relevance. The unchanged syrinxes at 3 months should also be interpreted cautiously, because syrinx regression may lag behind clinical improvement. However, persistent lack of radiological improvement may indicate a risk for delayed failure and requires longer serial MRI follow-up.

The surgical techniques in this study are largely consistent with those described in the literature [10,19,20,21]. However, one technical nuance in the current series was related to the timing of fibrin sealant application. As fibrin sealants require a period of undisturbed polymerization [22], we preferred to return the head and neck to the intended final neutral position before applying the sealant. This may allow the material to be set under definitive dural geometry and tissue tension, rather than under temporary conditions created by flexion. By contrast, application while the neck remains flexed may result in altered tissue tension once the head is brought back to a neutral position, which could affect the integrity of the barrier. Although this point has not been specifically addressed in the literature, it could represent a reasonable technical modification based on the biomechanical behavior of fibrin sealants. However, this maneuver was intended to optimize fibrin sealant application under the final tissue geometry, rather than to serve as a standalone measure for preventing CSF-related complications. The relatively high pseudomeningocele rate in the endoscopic group was probably multifactorial; factors such as onlay grafting, incomplete arachnoid preservation, and limited deep fascial closure through the endoscopic corridor may have reduced the protective effect of this maneuver. Beyond this technical point, intraoperative Doppler ultrasonography has potential value in demonstrating CSF flow, a point that has been relatively underemphasized in the literature.

CSF-related complications remain an important concern after duraplasty, particularly in series using onlay grafting, in which pseudomeningoceles and related complications have often been reported at relatively higher rates [23,24,25,26]. Recent randomized evidence has further highlighted the contemporary relevance of duraplasty in pediatric CM-I with syringomyelia, particularly regarding syrinx reduction and repeat decompression rates [27]. In the present study, duraplasty in the endoscopic group was performed with synthetic onlay grafts. Given the limited sample size, these events may have appeared proportionally more frequently; similarly, high rates have also been described in other small series [28,29,30,31,32,33]. Additionally, the definition of pseudomeningocele was deliberately inclusive; clinically evident cases as well as all radiologically detected collections were recorded, even when asymptomatic. Accordingly, although the overall complication profile did not differ significantly between the groups, pseudomeningocele was numerically more frequent in the endoscopic cohort than in the conventional cohort.

Technical explanations may explain these findings. In the conventional group, the dura was repaired using suturing, whereas the graft was placed as an onlay and reinforced with a fibrin sealant in the endoscopic group. In the initial cases of the endoscopic experience, endoscopic suturing of the dural graft was attempted; however, passage of the needle through the dura could create unintended arachnoid tears, and the limited depth perception and narrow working corridor make watertight suturing technically difficult. Such arachnoid violations may predispose the patient to a CSF fistula. Therefore, preserving the arachnoid whenever possible and avoiding full CSF egress during the dural opening are more reasonable strategies. However, this is not always technically achievable through an endoscopic corridor, and complete arachnoid preservation cannot be guaranteed in every case.

Historically, large dural defects encountered during spinal endoscopic procedures were commonly managed by conversion to an open approach to obtain a more secure closure [34,35,36,37]. More recently, however, several endoscopic dural repair techniques have been described, including full-endoscopic suturing, double-arm suture techniques, and non-penetrating clip-based closure [34,35,38,39]. These reports also emphasize the inherent limitations of endoscopic suturing. Unequal tension on the suture limbs may weaken knot security, and the passage of the needle through the dura may create additional needle holes that can become potential sites of persistent CSF leakage [35]. Accordingly, when primary closure is incomplete or when persistent leakage through suture holes is suspected, reinforcement with a dural sealant or other adjunctive material has been recommended [35,40]. Although these techniques were developed mainly in spinal endoscopic surgery and cannot be directly extrapolated to posterior fossa duraplasty, they support the same principle observed in the present series: achieving reliable dural closure through a narrow endoscopic corridor remains technically demanding and may contribute to postoperative pseudomeningocele formation.

Another factor may be related to the wound closure. Although the skin incision is short in the endoscopic approach, the muscle and fascial openings often extend beyond the skin margins to allow for adequate superior and inferior reach, respectively. Consequently, watertight reapproximation of deeper layers through such a limited skin window may be more difficult than in the conventional approach. This technical limitation may have contributed to the higher rate of pseudomeningoceles in the endoscopic group. Simultaneously, the small skin incision itself may protect against overt wound breakdown, which may partly explain why this did not translate into a clear increase in the number of external CSF fistulas [5,10].

Although no conversion was required in the present series, conversion to wider microscopic exposure may be considered in cases of uncontrolled bleeding, marked arachnoid disruption, high-flow CSF leakage, or inability to achieve acceptable dural reconstruction through the endoscopic corridor. In contrast, when CSF leakage is limited, even if a radiological pseudomeningocele develops, it may not necessarily result in a clinically significant CSF leak [31,33]. Lin et al. also described a decompression with an intradural dissection technique in which the dura was intentionally left open, and the resulting pseudomeningocele was regarded as a “neo-cisterna magna,” reporting no CSF wound fistula or symptomatic pseudomeningocele in their pediatric series [41]. Although their technique differs from the technique used in this study, this concept supports the view that not every postoperative CSF collection should be interpreted as a treatment failure.

Despite the higher pseudomeningocele rate, the rates of CSF fistula and wound infection in the endoscopic group were comparable to and numerically lower than those in the conventional group. Although a smaller incision may offer some advantages with respect to wound infection, reduced postoperative discomfort, and the perception of a less invasive procedure, it may have led some patients to underestimate early wound-related symptoms after discharge. However, no significant differences were found between the groups in either complication rate.

The endoscopic approach has several practical advantages. First, because the light source is located at the tip of the endoscope, it provides strong illumination deep in the operative corridor and allows wider and more panoramic visualization of anatomical details despite the narrow working space [10]. Additionally, surgery in the prone position can create ergonomic difficulties for surgeons during conventional decompression [42]. This may be particularly relevant in patients with a less favorable occipital angle, in whom the occipital bone may remain relatively vertical to the floor despite neck flexion, thus making the surgical trajectory more demanding. In such cases, previous studies have proposed modifying the skin incision cranially or caudally according to the occipital configuration [43]. However, with an endoscope, the viewing angle can be adjusted by tilting the scope itself rather than by forcing the surgeon to alter their body position or line of sight; in some of the present cases, angled endoscopes were used to overcome this anatomical difficulty. Another important ergonomic benefit of endoscopic surgery is the heads-up working posture, in which the surgeon operates while looking at a monitor directly in front, which can reduce physical fatigue [44].

Simultaneously, one disadvantage of endoscopy in Chiari surgery is the limited working space, which can make bimanual manipulation more difficult, particularly when using a 2-dimensional image setting. In a tubular or retractor-based corridor, both the endoscope and surgical instruments must enter through nearly the same axis, which may lead to instrument crowding and restricted freedom of movement [45]. Another practical limitation is the frequent soiling of the lens or screening with blood in endoscope-assisted systems that are not fully endoscopic, which may interrupt the flow of surgery and require repeated cleaning. Therefore, although endoscopy improves visualization and ergonomics in selected situations, it introduces technical constraints that should be considered when choosing the most appropriate approach. In our experience, the endoscope-assisted approach was not preferred in cases with recurrent Chiari-related adhesions, concomitant lesions such as tumors, or anticipated extensive intradural maneuvers such as tonsillar reduction. Because these situations often require wider exposure and bimanual three-dimensional microsurgical dissection, we believe that conventional surgery remains the more appropriate approach. Similarly, patients with C1–C2 instability requiring fixation were not considered suitable for this technique, because instrumentation requires a different surgical strategy rather than a limited endoscopic decompression corridor.

This study has several limitations, including its retrospective single-center design, relatively small sample size, use of a historical control cohort, and limited short-term follow-up. Therefore, the findings should be interpreted cautiously and confirmed in larger prospective studies. Although exploratory adjusted analyses were performed, residual confounding cannot be excluded. Another limitation is the short follow-up period. The 3-month assessment is insufficient to exclude delayed clinical failure or late syrinx progression, particularly in patients with unchanged syrinxes who require longer serial MRI follow-up.

Overall, the endoscope-assisted approach may provide a less-invasive surgical corridor while maintaining comparable short-term clinical and radiological outcomes. Although differences in cisterna magna expansion were observed, these did not translate into a clinical disadvantage, suggesting that the restoration of CSF flow, rather than the extent of decompression, may be more relevant. The technical nuances and limitations of the endoscopic technique should be considered; however, it represents a reasonable alternative in selected cases.

## 5. Conclusions

Endoscope-assisted PFDD may represent a less invasive alternative to conventional techniques, with comparable clinical and radiological outcomes. Although certain technical challenges remain, particularly those related to working within a limited corridor, it can be safely applied to selected patients. Further studies may help better define its role and optimize surgical techniques.

## Figures and Tables

**Table 1:** Preoperative demographic, clinical, and radiological characteristics by surgical approach.

Variable	Conventional (*n* = 16)		Endoscopic (*n* = 22)		*p*	SMD
	*n*	%	*n*	%		
**Age (years), mean ± SD**	35.4 ± 9.1		30.6 ± 18.9		0.303	−0.23
**Sex**					0.086	0.58
Female	9	56.3	18	81.8		
Male	7	43.8	4	18.2		
**Preoperative Clinical Symptoms**						
Headache (suboccipital, precipitated by Valsalva maneuver)	8	50	17	77.3	0.08	0.59
Neck/occipital pain	8	50	19	86.4	0.015	0.85
Motor deficit	7	43.8	3	13.6	0.037	−0.71
Sensory deficit	8	50	9	40.9	0.578	
Other symptoms (e.g., tinnitus, sleep-disordered breathing, shoulder/interscapular pain, autonomic dysfunction)	11	68.8	11	50	0.248	
Upper motor neuron signs	4	25	2	9.1	0.184	
Brainstem/cranial nerve involvement	3	18.8	4	18.2	0.964	
Cerebellar involvement	5	31.3	7	31.8	0.97	
**Preoperative Radiological Findings**						
**Syrinx**	8	50	12	54.5	0.782	0.09
Syrinx location					0.727	
None	8	50	10	45.5		
Cervical	5	31.3	5	22.7		
Cervicothoracic	2	12.5	6	27.3		
Holocord	1	6.3	1	4.5		
**Tonsillar descent level**					*—*	
Above C1	6	37.5	6	27.3		
At C1	6	37.5	12	54.5		
Below C1, not reaching C2	4	25	4	18.2		
**Osseous anomaly**					*—*	
None	16	100	17	77.3		
Only Platybasia	0	0	3	13.6		
Platybasia + Scoliosis	0	0	1	4.5		
Only Scoliosis	0	0	1	4.5		
**Preoperative cisterna magna**					0.647	0.15
None	12	75	15	68.2		
Minimal	4	25	7	31.8		
**Preoperative obex visibility**	7	43.8	17	77.3	0.034	0.73

*n*, number of patients; %, percentage; SD, standard deviation. Continuous variables are presented as mean ± SD; binary variables show only the number of patients with the feature present. —, *p*-value not computed (insufficient variation in one group). *p*-values were calculated using Welch’s independent-samples *t*-test for continuous variables and Fisher’s exact test or Pearson’s chi-square test for categorical variables. SMD, standardized mean difference; absolute SMD values ≥ 0.50 indicate moderate-to-large baseline imbalance.

**Table 2:** Comparison of postoperative outcomes, surgical variables, and complications between conventional and endoscopic surgical groups.

Variable	Conventional (*n* = 16)	Endoscopic (*n* = 22)	*p*
**Clinical Outcomes**			
Clinical improvement			0.952
No improvement	1 (6.3)	1 (4.5)	
Mild	2 (12.5)	2 (9.1)	
Moderate	5 (31.3)	6 (27.3)	
Marked	8 (50.0)	13 (59.1)	
Overall recovery	13 (81.3)	19 (86.4)	0.67
Postoperative MRI syrinx status			0.221
No syrinx	7 (43.8)	10 (45.5)	
Disappeared	2 (12.5)	0 (0.0)	
Shrunk	5 (31.3)	7 (31.8)	
Unchanged	1 (6.3)	5 (22.7)	
New syrinx formation	1 (6.3)	0 (0.0)	
Postoperative cisterna magna			**0.014**
None	1 (6.3)	6 (27.3)	
Minimal	3 (18.8)	10 (45.5)	
Sufficient	12 (75.0)	6 (27.3)	
Chicago Chiari Outcome Scale (CCOS), at discharge	11.4 ± 2.2	13.2 ± 2.0	**0.017**
Chicago Chiari Outcome Scale (CCOS), at 3 months	13.4 ± 1.8	14.0 ± 1.5	0.265
**Surgical Variables**			
Incision length (cm), mean ± SD	6.06 ± 0.83	2.45 ± 0.50	<**0.001**
Ambulatory day, mean ± SD	1.5 ± 0.8	0.5 ± 0.5	<0.001
Length of hospital stay (days), mean ± SD	5.2 ± 2.0	3.0 ± 3.0	**0.013**
**Postoperative Complications**			
Pseudomeningocele	3 (18.8)	10 (45.5)	0.165
CSF fistula	4 (25.0)	4 (18.2)	0.698
Lumbar drainage	3 (18.8)	1 (4.5)	0.291
Wound infection	3 (18.8)	4 (18.2)	1
Subdural/epidural hematoma	0 (0.0)	0 (0.0)	

Values are presented as *n* (%) or mean ± SD. Bold *p* values indicate statistical significance (*p* < 0.05). CCOS, Chicago Chiari Outcome Scale (range 4–16; higher scores indicate better outcome). Binary symptom variables show only the number of patients with the feature present.

## Data Availability

The data supporting the findings of this study are available from the corresponding author upon reasonable request.

## Supplementary Materials

The following supporting information can be downloaded at: https://doi.org/10.5281/zenodo.20272378, Video S1: Surgical demonstration of the endoscopic posterior fossa decompression technique; https://www.mdpi.com/article/10.3390/medicina62071285/s1, Table S1: Exploratory adjusted sensitivity analyses; Table S2: Postoperative complications, unadjusted analysis; Table S3: Syrinx outcome among patients with preoperative syringomyelia.

## Abbreviations

CM-I	Chiari malformation type I
CSF	cerebrospinal fluid
PFD	posterior fossa decompression
PFDD	posterior fossa decompression with duraplasty
MRI	magnetic resonance imaging
CCOS	Chicago Chiari Outcome Scale
SD	standard deviation
CT	computed tomography
SMD	standardized mean difference
