# Supplementary material for: Endoscope-Assisted Versus Conventional Posterior Fossa Decompression with Duraplasty for Chiari I Malformation: A Single-Center Comparative Study
Source: Medicina (Kaunas). 2026 Jul 3;62(7):1285. doi: 10.3390/medicina62071285 (PMC13414425; doi:10.3390/medicina62071285)
Supplement: Supplementary file 1 [file medicina-62-01285-s001.zip › medicina-4355900-supplementary.pdf]

Supplementary Material

Endoscopic versus conventional posterior fossa decompression with duraplasty (PFDD) for Chiari I malformation (conventional n = 16, endoscopic n = 22; total n = 38).

Supplementary Table S1. Exploratory adjusted sensitivity analyses for the association between surgical approach and postoperative outcomes

Independent variable of interest: surgical approach (reference = conventional PFDD; estimate = effect of the endoscopic approach). Main adjustment set: age, sex, preoperative motor deficit, and syrinx presence. Continuous outcomes were modeled by linear regression with HC1 robust standard errors and verified with 5,000-replication bootstrap percentile confidence intervals. Selected binary outcomes included in Supplementary Table S1 were modeled with Firth penalized logistic regression to mitigate small-sample bias and separation.

Outcome	Model	Covariates	Adjusted estimate (endoscopic)	95% CI	p
CCOS at discharge	Linear, robust SE	Approach + age + sex + motor + syrinx	β = +1.91	0.08 to 3.74 (boot 0.22 to 4.01)	0.041
CCOS at 3 months	Linear, robust SE	Approach + age + sex + motor + syrinx	β = +0.93	−0.43 to 2.29 (boot −0.34 to 2.47)	0.182
Overall recovery (yes/no)	Firth logistic	Approach + age + sex + motor + syrinx	aOR = 1.99	0.29 to 13.58	0.482
Ambulatory day	Linear, robust SE	Approach + age + sex + motor + syrinx	β = −0.95 day	−1.48 to −0.41 (boot −1.57 to −0.45)	0.001
Ambulatory day (sensitivity)	Poisson, robust SE	Approach + age + sex + motor + syrinx	IRR = 0.39	0.24 to 0.63	<0.001
Postoperative cisterna magna	Firth logistic	Approach + preop cisterna	aOR = 0.18	0.04 to 0.81	0.025

(sufficient vs none/minimal)		magna + motor			

Estimates express the effect of the endoscopic approach relative to conventional PFDD. β, adjusted regression coefficient; aOR, adjusted odds ratio; IRR, incidence rate ratio; CI, confidence interval; boot, bootstrap percentile 95% CI (5,000 replications); CCOS, Chicago Chiari Outcome Scale.

A negative β for ambulatory day and an aOR < 1 for sufficient cisterna magna both reflect the direction of the endoscopic effect (earlier ambulation; less frequent sufficient cisterna magna formation).

Given the small sample (n = 38), these analyses are exploratory and hypothesis-generating, with a substantial risk of residual confounding and overfitting; no superiority or causal claim is intended.

Supplementary Table S2. Postoperative complications, unadjusted analysis

Multivariable models were not fitted because of sparse events. Two-sided Fisher exact tests were used for between-group comparisons, and unadjusted odds ratios with 95% confidence intervals were reported.

Complication	Endoscopic n/N (%)	Conventional n/N (%)	OR (95% CI)	Fisher p
Pseudomeningocele	10/22 (45.5)	3/16 (18.8)	3.61 (0.80–16.35)	0.165
CSF fistula	4/22 (18.2)	4/16 (25.0)	0.67 (0.14–3.19)	0.698
Lumbar drainage	1/22 (4.5)	3/16 (18.8)	0.21 (0.02–2.20)	0.291
Wound infection	4/22 (18.2)	3/16 (18.8)	0.96 (0.18–5.06)	1.000

Odds ratios express the odds of each complication in the endoscopic group relative to the conventional group. All patients who received lumbar drainage had a CSF leak (lumbar drainage represents the subset of CSF leaks managed with a lumbar drain). These p values are consistent with the complication comparisons reported in Table 2 of the main text.

Supplementary Table S3. Syrinx outcome among patients with preoperative syringomyelia

Restricted to patients with a preoperative syrinx (endoscopic n = 12, conventional n = 8). Outcome was dichotomized as improved, defined as disappeared/resolved or

reduced/shrunk, versus unchanged. Owing to small numbers, no multivariable model was fitted; unadjusted Fisher exact test and unadjusted Firth logistic regression are reported.

Analysis	Improved — Endoscopic	Improved — Conventional	OR (95% CI)	p
Fisher exact	7/12 (58.3%)	7/8 (87.5%)	0.27 (0.03–2.16)	0.325
Firth logistic (unadjusted)	—	—	0.27 (0.03–2.42)	0.244

Exploratory; not a primary endpoint.

Supplementary methods

Standardized mean differences were computed to assess baseline balance between groups. For postoperative outcomes, continuous variables were analyzed by linear regression with robust standard errors and verified by bootstrap confidence intervals. Selected binary outcomes were analyzed with Firth penalized logistic regression. Complications and syrinx outcomes were analyzed unadjusted because of sparse events. Given the limited sample size, all adjusted analyses are exploratory and hypothesis-generating. All analyses used conventional PFDD as the reference category, and effect estimates express the effect of the endoscopic approach; no superiority or causal claim is intended.
